# Identification of immune-related genes and small-molecule drugs in hypertension-induced left ventricular hypertrophy based on machine learning algorithms and molecular docking

**DOI:** 10.3389/fimmu.2024.1351945

**Published:** 2024-06-27

**Authors:** Mingxuan Zhou, Tiegang Li, Silin Lv, Wenqiang Gan, Fang Zhang, Yuexia Che, Liu Yang, Yufang Hou, Zheng Yan, Zifan Zeng, Wenyi Zhao, Min Yang

**Affiliations:** ^1^ State Key Laboratory of Bioactive Substances and Function of Natural Medicine, Institute of Materia Medica, Chinese Academy of Medical Sciences and Peking Union Medical College, Beijing, China; ^2^ School of Pharmacy, Minzu University of China, Beijing, China

**Keywords:** left ventricular hypertrophy, hypertension, RNA sequencing, immune response, machine learning, molecular docking

## Abstract

**Background:**

Left ventricular hypertrophy (LVH) is a common consequence of hypertension and can lead to heart failure. The immune response plays an important role in hypertensive LVH; however, there is no comprehensive method to investigate the mechanistic relationships between immune response and hypertensive LVH or to find novel therapeutic targets. This study aimed to screen hub immune-related genes involved in hypertensive LVH as well as to explore immune target-based therapeutic drugs.

**Materials and methods:**

RNA-sequencing data from a mouse model generated by angiotensin II infusion were subjected to weighted gene co-expression network analysis (WGCNA) to identify core expression modules. Machine learning algorithms were applied to screen immune-related LVH characteristic genes. Heart structures were evaluated by echocardiography and cardiac magnetic resonance imaging (CMRI). Validation of hub genes was conducted by RT-qPCR and western blot. Using the Connectivity Map database and molecular docking, potential small-molecule drugs were explored.

**Results:**

A total of 1215 differentially expressed genes were obtained, most of which were significantly enriched in immunoregulation and collagen synthesis. WGCNA and multiple machine learning strategies uncovered six hub immune-related genes (*Ankrd1, Birc5, Nuf2, C1qtnf6, Fcgr3, and Cdca3*) that may accurately predict hypertensive LVH diagnosis. Immune analysis revealed that fibroblasts and macrophages were closely correlated with hypertensive LVH, and hub gene expression was significantly associated with these immune cells. A regulatory network of transcription factor-mRNA and a ceRNA network of miRNA-lncRNA was established. Notably, six hub immune-related genes were significantly increased in the hypertensive LVH model, which were positively linked to left ventricle wall thickness. Finally, 12 small-molecule compounds with the potential to reverse the high expression of hub genes were ruled out as potential therapeutic agents for hypertensive LVH.

**Conclusion:**

This study identified and validated six hub immune-related genes that may play essential roles in hypertensive LVH, providing new insights into the potential pathogenesis of cardiac remodeling and novel targets for medical interventions.

## 1 Introduction

Left ventricular hypertrophy (LVH) occurs when cardiac muscle adapts to elevated blood pressure, manifesting as increased thickness of the left ventricular wall (1). A common outcome of hypertension end-organ damage, LVH is linked to increased morbidity and mortality of cardiovascular disease since it often causes heart failure, stroke, and arrhythmias. The physiopathology of LVH involves complex interactions between cardiomyocytes and cardiac non-myocytes, including endothelial cells, fibroblasts, and the immune system. However, the molecular mechanism underlying LVH remains incompletely understood, and LVH treatments are limited. Identifying the regulators and altered mechanisms of hypertrophy is essential for the development of treatments to curtail the progression of LVH.

Solid evidence has indicated the critical role of immune activity in the development of cardiac remodeling (2–5). The presence of resident and recruited immune cells in the heart precedes hypertrophy and results in cardiac dysfunction and failure. The accumulation of macrophages in the left ventricular wall through both local proliferation and monocyte recruitment has been proved to be linked to LVH. It has been found that the cardiac macrophage population grows before visible hypertrophy, which implies that inflammatory changes participate in the development of LVH. Our team previously proved that inhibiting the activation of the nucleotide-binding domain, leucine-rich–containing family, pyrin domain–containing-3 (NLRP3) inflammasome of macrophages prevents angiotensin II (ANG II) -induced cardiac inflammation and fibrosis (6). Furthermore, the depletion of macrophages attenuates LVH by decreasing diastolic and systolic wall thickness (7, 8). In addition to macrophages, immune activity also involves fibroblasts during cardiac remodeling. Quiescent cardiac fibroblasts change into their active forms because of cytokines, chemokines, and growth factors, and consequently, the increased paracrine factors from fibroblasts recruit immune cells and provoke acute inflammatory changes (9). Considering the critical role and complexity of the immune response in the pathophysiology of ventricular remodeling, systematic and comprehensive analyses of immune-related genes contributing to LVH and novel therapeutic targets are urgently needed.

Recently, the development of RNA-seq and bioinformatics has made it possible to elucidate the underlying mechanisms of diseases. In the field of cancer, key biomarkers have been well mined and studied through bioinformatic methods (10). As for hypertension, some studies have aimed to identify relevant differentially expressed genes (DEGs) and hub genes. A previous study identified three hub genes, such as BH3 interacting domain death agonist (*BID*), in limited cutaneous systemic sclerosis-associated pulmonary arterial hypertension (11). However, there has been little investigation into the molecular targets of hypertension-induced LVH. In this study, we sequenced the left ventricles of ANG II-infusing mice and explored the immune cell profile using multiple algorithms. Subsequently, we identified the immune-related module through WGCNA and screened the hub genes with machine learning algorithms. Additionally, small-molecule compounds with the potential to serve as therapeutic drugs for hypertensive LVH treatment were identified.

## 2 Materials and methods

### 2.1 Animal preparation and sample collection

Male wild-type C57BL/6J mice (Beijing Vital River Laboratory Animal Technology Company, Beijing, China) were maintained under specific pathogen-free conditions. Mice were infused for 7 days with saline or a “pressor dose” of ANG II (1500 ng/kg/min) by osmotic mini-pumps (Alzet MODEL 1007D; DURECT, Cupertino, CA, USA) implanted subcutaneously as previously described (12). Cardiac magnetic resonance imaging (CMRI) was performed using a PharmaScan 70/16 US (7.0 T, Bruker, Switzerland) after 7 days of ANG II or saline infusion as previously described (13). Echocardiography was performed using a Vevo 770 High-Resolution Imaging System (VisualSonics Inc). All measurements were averaged over five consecutive cardiac cycles and interpreted by an experienced technician blinded to the treatment group.

**Figure 1:**
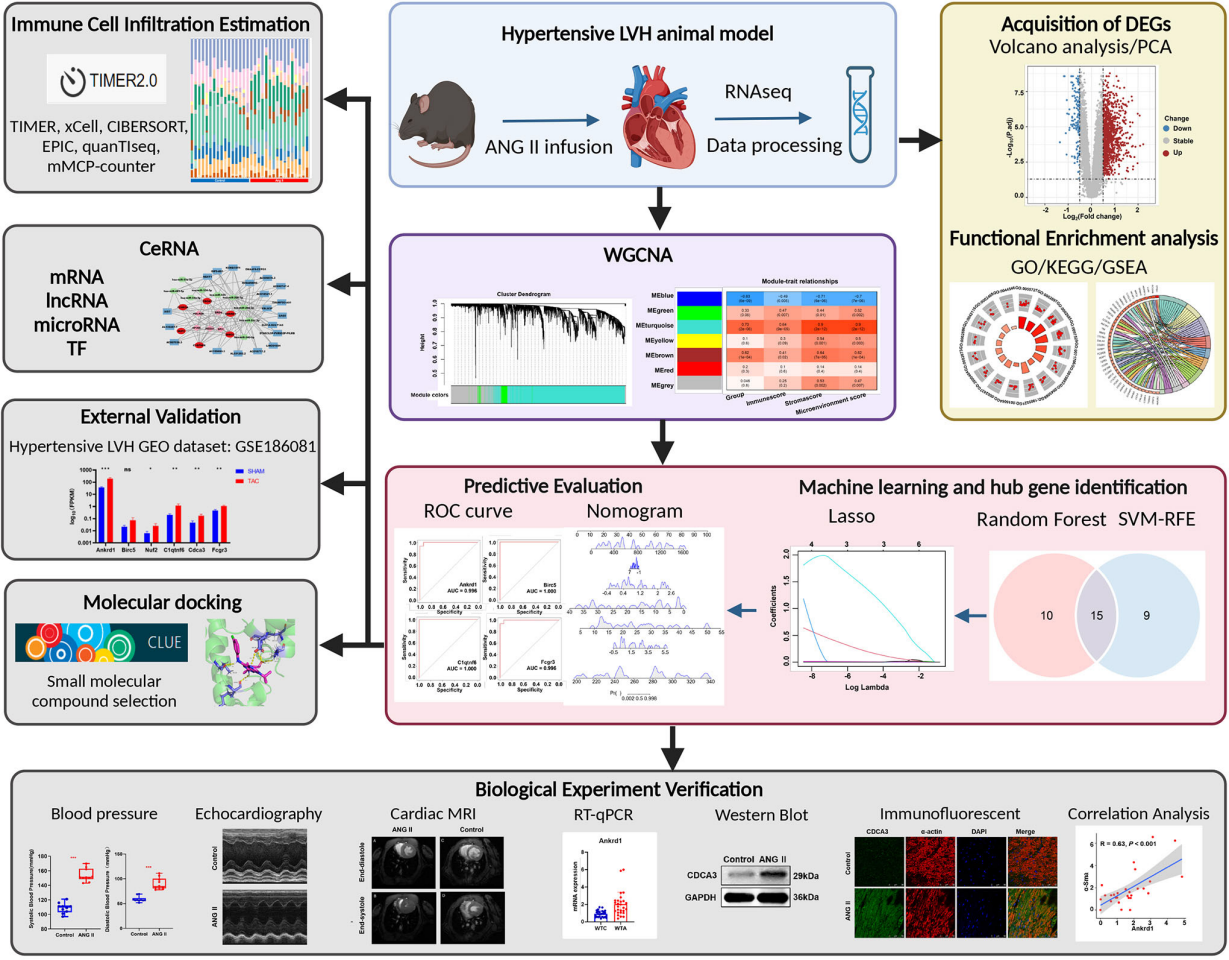
Flowchart of this study.

Mice were anesthetized following experimental treatment, and then the heart was punctured and flushed with 20 ml of saline to remove blood from systemic circulation. The left ventricles were removed and prepared for further RNA-seq analyses. The entire procedure of this study is shown in **Figure 1**.

### 2.2 RNA-seq

RNA-seq RNA extraction, quality control, sample preparation, library construction, and sequencing technologies were provided by Beijing ECOBID Technology Company. The sequencing samples included control and hypertensive-LVH groups, each with 16 biological replicates. The samples were tested by Nanodrop for RNA concentration, OD260/280, OD260/230, and Agilent 2100 for RNA fragment length. Then, mRNA was enriched using Oligo (dT) magnetic beads, and the enriched mRNA was interrupted and split into two pieces. After enrichment, the mRNA was broken into fragments and synthesized into one-stranded cDNA by adding six-base random primers, and then two-stranded cDNA was synthesized by adding buffer, dNTPs, and DNA polymerase I. The synthesized double-stranded cDNA was purified, end-repaired, A-added, spliced, and screened for fragment size by using AMPure XP beads. Finally, a cDNA library was constructed by polymerase chain reaction (PCR) amplification.

The constructed cDNA library was analyzed by quantitative PCR (qPCR) to determine the effective concentration of the library, and by Agilent 2100 to determine the size of the inserted fragments of the library. The cDNA library was pooled according to the effective concentration from the library and data requirements and sequenced on the computer. Illumina’s second-generation high-throughput sequencing platform and PE150 double-end sequencing method were used.

Additionally, eight samples in GSE186081 (14) of the SHAM-LVH group and the transverse aortic constriction-LVH (TAC-LVH) group were included for external validation. In GSE186968 (15), six samples at postnatal day 14 were used for volume overload analysis of LVH.

### 2.3 Differential analysis and pathway enrichment analysis

Principle component analysis (PCA) was conducted on RNA-seq data using the R package “scatterplot3d”. The R packages “limma” and “egdeR” were used to determine DEGs between the control group and the hypertensive-LVH group, with the thresholds of adjusted *P* < 0.05 and absolute value of log [fold change (FC)] > 0.5. The Kyoto Encyclopedia of Genes and Genomes (KEGG) pathway and Gene Ontology (GO) analyses were performed using the gene names, and Gene Set Enrichment Analysis (GSEA) was conducted with the gene names and log (FC) between the phenotype labels “control” and “ANG II.” All these analyses were conducted in the R package “clusterProfiler”. A comprehensive presentation of enriched pathways was presented by Metascape, and the pathway enrichment was conducted in Metascape (http://metascape.org/) as well.

### 2.4 Immune infiltration estimation analysis

The level of immune infiltration in each sample was tested using six methods: Tumor Immune Estimation Resource (https://cistrome.shinyapps.io/timer/, TIMER) (16, 17), xCell (18), Cell-type Identification By Estimating Relative Subsets of RNA Transcripts (CIBERSORT) (19), the murine Microenvironment Cell Population counter (mMCP-counter) (20), the quantification of the Tumor Immune contexture from human RNA-seq data (quanTIseq) (21), and Estimating the Proportion of Immune and Cancer cells (EPIC) (22). The list of immune stimulators was extracted from the Tumor-Immune System Interaction Database (TISIDB) (23).

### 2.5 Weighted gene co-expression network analysis

Weighted Gene Co-expression Network Analysis (WGCNA) was conducted by the R package “WGCNA” to identify key modules that correlated with the immune system. First, cases were categorized by hierarchical clustering. Analysis of scale independence and mean connectivity was carried out to filter the optimal soft power β using the pickSoftThreshold R function; this allowed for the better detection of strong correlations between gene modules. Then, gene modules were built. The relationships between modules and experimental groups, immune score, stroma score, and microenvironment score of xCell were explored. The module most connected with the traits was selected, and the scatterplot of module membership and gene significance was drawn.

### 2.6 Identification of hub genes by machine learning

Genes in the target module were then tested by support vector machine recursive feature elimination (SVM-RFE) through the R package “caret” and by the random forest algorithm. The intersection of the hub genes obtained by the two algorithms was then screened by least absolute shrinkage and selection operator (LASSO) regression through the R package “glmnet”. The Venn diagram was drawn by the R package “ggvenn.”

### 2.7 Construction of competitive endogenous RNA network

To probe the factors associated with the function of hub genes, we constructed a competitive endogenous RNA network. The ENCORI database (24) was used to obtain the miRNA families that bound to six hub genes. Nine miRNAs that were related to all hub genes were selected, and the corresponding lncRNAs and miRNA bound to the selected miRNAs were obtained for further investigation. Additionally, we utilized hTFtarget (25) to identify transcription factors (TFs) correlated with immune-related hub genes. Then, a regulatory network of transcription factor-mRNA-miRNA-lncRNA interactions was constructed using Cytoscape software (version 3.9.1, U.S. National Institute of General Medical Sciences).

### 2.8 Real-time quantitative PCR

The total RNA of experimental samples was extracted using an RNeasy kit (Beyotime, Shanghai, China, R0027) in accordance with the manufacturer’s instructions. Then, we reverse-transcribed 1 μg of total RNA using SuperScript II reverse transcriptase (Takara, Japan, RR047). Finally, RT-qPCR analysis was performed using SYBR Green Master Mix (Takara, Japan, RR820) in an ABI 7900 HT real-time PCR system. The primer sequences for RT-qPCR are listed in **Supplementary Table 1**.

### 2.9 Histology and immunofluorescence staining

Samples of cardiac tissue were fixed in 4% paraformaldehyde, dehydrated, embedded in paraffin, and sliced into 5-μm slides. Masson’s trichrome staining was carried out according to established protocols. Using ImageJ, interstitial fibrotic areas were determined; here, they were defined as the ratio of the total area of interstitial fibrosis to the total section area.

For IF staining, cardiac sections were incubated overnight with primary antibodies for α-Actin (A5044, SIGMA, 1:200, mouse), Nuf2 (bs-7714R, Bioss, 1:100, rabbit), and Cdca3 (bs-7894R, Bioss, 1:100, rabbit). Then, these sections were incubated with secondary antibodies, anti-rabbit (bs-0295G-AF488, Bioss, 1:100) and anti-mouse IgG (115–625-205, Jackson, 1:200), followed by staining with 4’,6-diamidino-2-phenylindole (DAPI) and mounting with glycerol before imaging via confocal laser scanning microscopy (Leica, Wetzlar, Germany). FITC-conjugated wheat germ agglutinin (WGA, W11261, Invitrogen, 1:200) was used to evaluate the cardiomyocyte cross-sectional area. Cell area determinations were based on measurements of at least 200 cells per slide.

### 2.10 Western blotting analysis

Total protein from cardiac tissue was collected using lysis buffer (Cwbio, Beijing, China) supplemented with protease inhibitor cocktail (Roche, Basel, Switzerland). Protein concentration was quantified through a bicinchoninic acid protein assay (Pierce Biotechnology, Rockford, IL, USA), after which the protein was separated by 10% or 12% sodium dodecyl sulphate-polyacrylamide gel electrophoresis and transferred onto nitrocellulose membranes (Bio-Rad, Hercules, CA, USA). Membranes were incubated with specific primary antibodies against Nuf2 (bs-7714R, Bioss, 1:500) and Cdca3 (bs-7894R, Bioss, 1:500, rabbit), and GAPDH (#5174, Cell Signaling, 1:1000) at 4°C overnight, followed by incubation with the appropriate secondary antibodies (S8002, Sudgen, 1:10000). Images were obtained using an ImageQuant™ LAS 4000 luminescent image analyzer (GE, Boston, MA, USA).

### 2.11 Screening of small-molecule compounds and molecular docking

DEGs were input into the Connectivity Map (CMap) website (https://clue.io/). CMap compares similarities in gene expression to screen for potential therapeutic compounds. A negative score implies that the drug has the potential to reverse pathological processes.

The structures of potential small-molecule drugs were collected from PubChem (https://pubchem.ncbi.nlm.nih.gov/). The 3D structures of hub proteins were downloaded from RCSB Protein Data Bank (PDB, http://www.rcsb.org/) and AlphaFold (https://alphafold.ebi.ac.uk/). The proteins were dehydrated and ligands extracted by PyMOL and saved in PDBQT format by the software AutoDock. The docking simulations were performed by AutoDock Vina. Finally, the drug-protein binding targets were visualized in PyMOL.

### 2.12 Statistical analysis

R software (version 3.2) and GraphPad Prism 8.0 (GraphPad Software Inc., San Diego, CA, USA) were used for data processing and visualization. Continuous variables were analyzed using the Wilcoxon rank-sum test or Kruskal–Wallis test. Spearman’s correlation analyses were used. The significance of large-scale multiple tests was evaluated by the Benjamini–Hochberg method.

## 3 Results

### 3.1 Identification of DEGs and pathway enrichment

**Figure 2:**
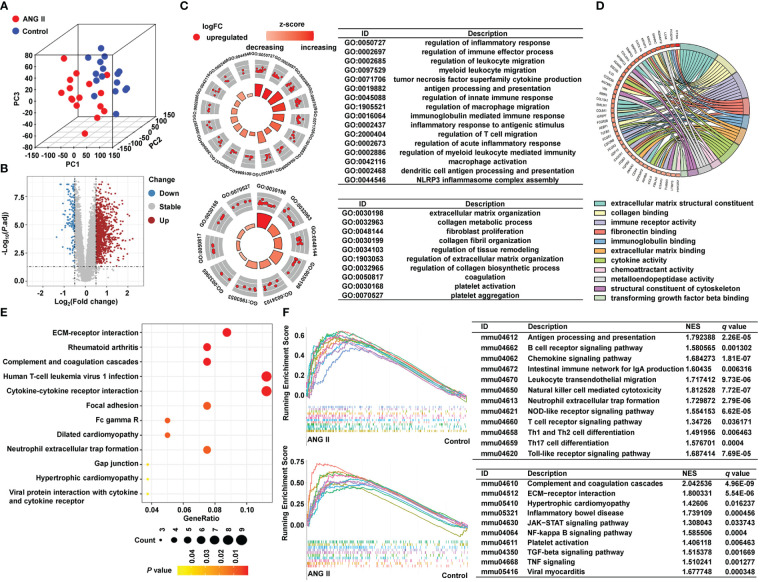
Identification of DEGs and pathway enrichment. **(A)** Three-dimensional principal component analysis. **(B)** A volcano plot showing 1215 differentially expressed genes. **(C)** Pathway enrichment result of GO biological process. **(D)** Pathway enrichment result of GO molecular function. **(E)** KEGG analysis. **(F)** GSEA result of KEGG. GO, Gene Ontology. KEGG, Kyoto Encyclopedia of Genes and Genomes. GSEA, Gene Set Enrichment Analysis.

We collected RNA-seq data from the control group and the ANG II-induced hypertensive-LVH group, with 16 samples from each group, and transferred the data into fragments per kilobase of transcript per million mapped reads (FPKM) format. A three-dimensional PCA plot was created to demonstrate the comparability between the two groups (**Figure 2A**). In total, 1215 DEGs were identified with the criteria of adjusted *P* value < 0.05 and absolute value of log (FC) > 0.5 (**Figure 2B**). In the hypertensive-LVH group, there were 1073 upregulated genes and 142 downregulated genes compared with the control group.

To examine the underlying mechanisms of the two groups, we conducted pathway enrichment analysis. As shown in **Figure 2C** and **Supplementary Figure 1**, biological process pathways enriched in the hypertensive-LVH group were mostly related to the immune system and extracellular matrix (ECM), such as regulation of immune effector process, regulation of macrophage migration, and ECM organization. The GO chord plot shows that genes upregulated in the hypertensive-LVH group were also enriched in immune response and ECM (**Figure 2D**). KEGG analysis revealed that pathways such as ECM-receptor interaction, human T-cell leukemia virus 1 infection, and cytokine-cytokine receptor interaction were enriched in the hypertensive-LVH group (**Figure 2E**). Moreover, dilated cardiomyopathy and hypertrophic cardiomyopathy were enriched. The upper part of **Figure 2F** shows that immune cell infiltration was engaged in the hypertensive-LVH group, which was determined by GSEA, and the lower part of the figure displays typical signaling pathways enriched in the hypertensive-LVH group, like the Janus kinase-signal transducer and activator of transcription (JAK−STAT) signaling pathway, nuclear factor kappa B (NF-κB) signaling pathway, and TGF-β signaling pathway. The pathway enrichment analysis indicated that immune-related activity was heightened in the hypertensive-LVH group.

### 3.2 Identification of hub genes by WGCNA and machine learning

**Figure 3:**
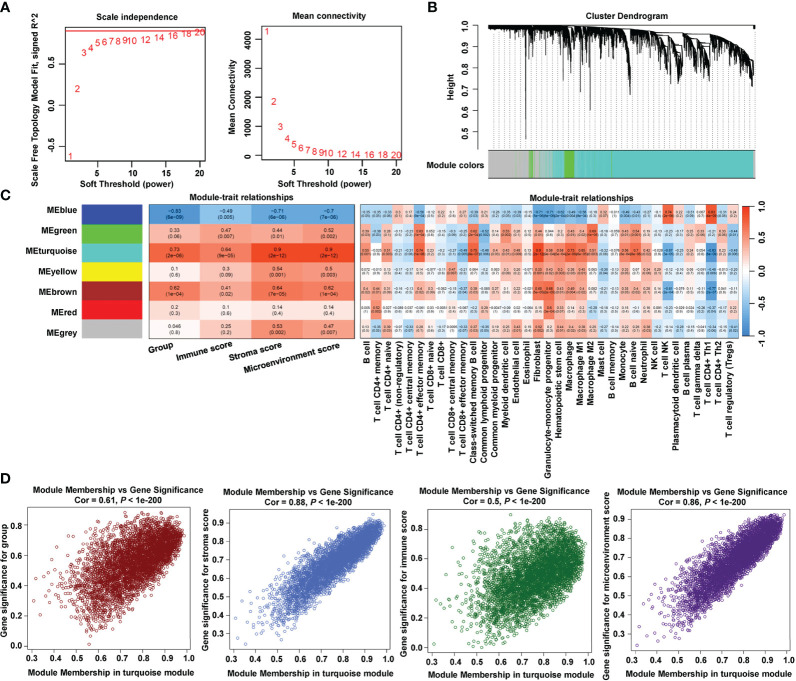
Identification of immune-related module by WGCNA. **(A)** Estimation of the scale independence index of the 1–20 soft threshold power (β = 18) and determination of the mean connectivity of the 1–20 soft threshold power. **(B)** Cluster dendrogram of gene co-expression. **(C)** Relationships between consensus module eigengenes and immune characteristics. **(D)** Relationships between module membership in turquoise module and gene significance.

To identify hub genes related to the immune process in LVH, we adopted WGCNA to find immune-related modules. First, the optimal soft power β = 18 was confirmed by scale independence and mean connectivity (**Figure 3A**). Among the seven co-expression modules identified (**Figure 3B**; **Supplementary Table 2**), module turquoise (4898 genes) had the strongest positive correlation with group, immune score, stroma score, and microenvironment score (**Figure 3C**). Additionally, fibroblast infiltration computed by xCell had the strongest correlation with module turquoise. Next, we specifically examined correlations between the module membership in turquoise and the gene significance for each trait (**Figure 3D**). The spearman’s correlation coefficient between the module membership and gene significance for group was 0.61, for immune score was 0.88, for stroma score was 0.5, and for microenvironment score was 0.86, all with *P* value < 1e-200. These findings showed that the turquoise module was the most connected to immune infiltration.

**Figure 4:**
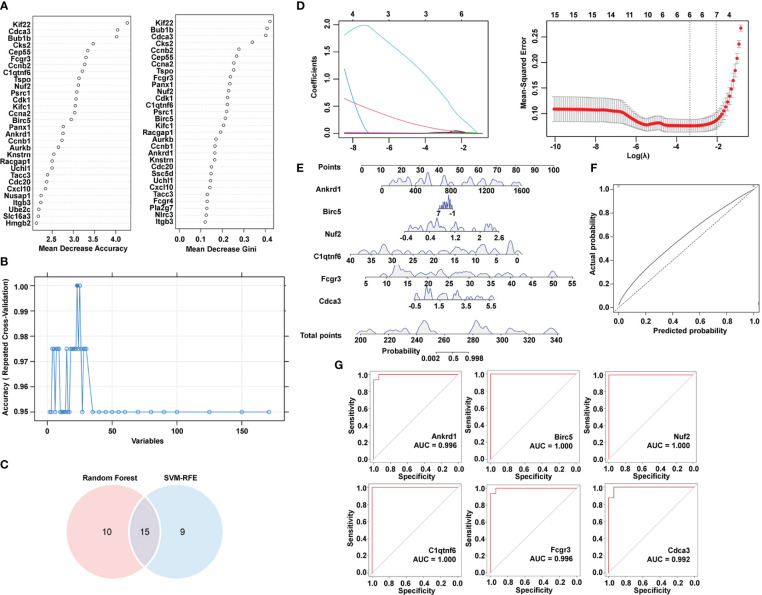
Identification of hub immune-related genes by machine learning algorithms. **(A)** Top 30 most important genes identified by mean decrease accuracy and mean decrease Gini of random forest. **(B)** SVM-RFE algorithm to determine hub genes. **(C)** Venn diagram showing the intersection of two algorithms. **(D)** LASSO regression to determine the hub genes. **(E)** Nomogram combining six hub genes screened by Lasso. **(F)** ROC curve of predicted score by nomogram. **(G)** ROC curve of each hub gene expression for LVH. SVM-RFE, support vector machine-recursive feature elimination. LASSO, least absolute shrinkage and selection operator. ROC, receiver operating characteristic. LVH, left ventricular hypertrophy.

SVM-RFE and random forest were utilized to downsize genes in module turquoise. **Figure 4A** shows the top 30 genes in mean decrease accuracy and mean decrease Gini calculated by random forest. Among them, 24 genes were identified as important genes distinguishing the control group from the hypertensive-LVH group (**Figure 4B**). A Venn diagram showed that there were 15 overlapping genes identified by two algorithms (**Figure 4C**), which were kinesin family member 22 (*Kif22*), cell division cycle associated 3 (*Cdca3*), mitotic checkpoint serine/threonine kinase (*Bub1b*), centrosomal protein 55 (*Cep55*), Fc receptor, IgG, low-affinity III (*Fcgr3*), C1q and tumor necrosis factor–related protein 6 (*C1qtnf6*), translocator protein (*Tspo*), NDC80 kinetochore complex component (*Nuf2*), cyclin A2 (*Ccna2*), baculoviral IAP repeat containing 5 (*Birc5*), pannexin 1 (*Panx1*), ankyrin repeat domain 1 (*Ankrd1*), cyclin B1 (*Ccnb1*), ubiquitin carboxy-terminal hydrolase L1 (*Uchl1*), and transforming, acidic coiled-coil containing protein 3 (*Tacc3*). Finally, LASSO regression recognized six hub genes with the lowest binominal deviance (**Figure 4D**). The hub genes were *Ankrd1*, *Birc5*, *Nuf2*, *C1qtnf6*, *Fcgr3*, and *Cdca3*. A nomogram was constructed to predict the occurrence of hypertension-indicated LVH (**Figure 4E**), and the receiver operating characteristic (ROC) curve was drawn to evaluate the accuracy of the nomogram (**Figure 4F**). Additionally, the ROC curve of each hub gene was drawn separately in **Figure 4G**. The area under the curve (AUC) of *Birc5*, *Nuf2*, and *C1qtnf6* was 1.000 and the AUC of *Ankrd1* and *Fcgr3* was 0.996. These results showed that we successfully identified six hub immune-related genes with the potential to diagnose between LVH and normal heart tissue.

### 3.3 Immune infiltration landscape and correlation with hub genes

**Figure 5:**
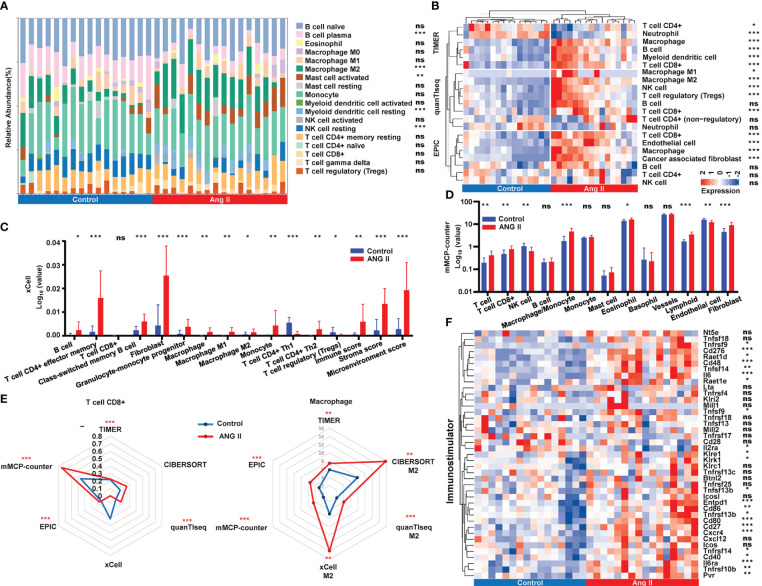
Immune infiltration landscape analysis. **(A)** Relative abundance of immune infiltration calculated by CIBERSORT. **(B)** Immune infiltration of immune cells obtained by TIMER, quanTIseq, and EPIC. **(C)** Comparison of immune cell abundance between control and ANG II based on xCell. **(D)** Comparison of immune cell abundance between control and ANG II based on mMCP-counter. **(E)** Radar plot demonstrating the extent of CD8+ T cells and M2 macrophages between the control group and the hypertensive-LVH group by six algorithms. **(F)** The expression of immunostimulatory genes by control and ANG II. CIBERSORT, Cell-type Identification by Estimating Relative Subsets of RNA Transcripts. TIMER, Tumor Immune Estimation Resource. quanTIseq, quantification of the Tumor Immune contexture from human RNA-seq data. EPIC, Estimating the Proportion of Immune and Cancer cells. ANG II, angiotensin II. mMCP-counter, murine Microenvironment Cell Population counter. ***P < 0.001; **P < 0.01; *P < 0.05; ns, not significantly.

It has been reported that immune cell interactions and inflammatory signaling mechanisms play crucial roles in cardiac hypertrophy and remodeling (26). We adopted six deconvolution algorithms to explore the immune infiltration distinction between the control group and the hypertensive-LVH group. The quantification of the relative proportion computed by CIBERSORT is shown in **Figure 5A**. Three algorithms, namely, TIMER, quanTIseq, and EPIC, found that macrophages and CD8+ T cells had higher levels of infiltration in the hypertensive-LVH group (**Figure 5B**). The results predicted by xCell showed that all the immune cells except CD8+ T cells, Th1 subset of CD4+ T cells, and regulatory T cells exhibited higher proportions in the hypertensive-LVH group and higher scores of the immunity, stroma, and microenvironment (**Figure 5C**). The prediction of mMCP-counter revealed that T cells, CD8+ T cells, macrophages, eosinophils, lymphoids, endothelial cells, and fibroblasts were significantly upregulated in the hypertensive-LVH group (**Figure 5D**). Specifically, the radar plot demonstrates that the relative proportions of CD8+ T cells and macrophages increased overall in the hypertensive-LVH group (**Figure 5E**). In addition, the expression of common immune stimulators was compared between the two groups, and genes such as *Cd276*, interleukin 6 (*Il6*), *Cd80*, *Cd27*, and C-X-C motif chemokine receptor (*Cxcr4*) were all significantly upregulated in the hypertensive-LVH group (**Figure 5F**; **Supplementary Figure 2**). The data demonstrated that in the hypertensive-LVH group, the proportions of macrophages, CD8+ T cells, and fibroblasts were higher than in the control group.

**Figure 6:**
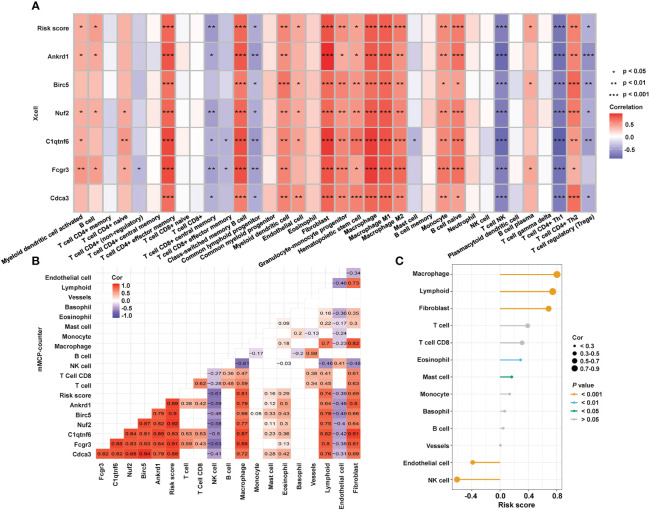
Relationship between hub genes and immune infiltration. **(A)** Correlations among the abundance of immune cells, the expression of hub genes, and risk score, as determined by xCell. **(B)** Correlations among the immune infiltration, the expression of hub genes, and risk score, as calculated by mMCP-counter. **(C)** Correlation between immune cells and risk score as computed by an mMCP-counter. mMCP-counter, murine Microenvironment Cell Population counter. ***P < 0.001; **P < 0.01; *P < 0.05.

To investigate the mechanistic relationship between hub genes and immune infiltration, we compared the expression of hub genes and immune proportion. According to xCell, effector memory CD4+ T cells, cancer associated fibroblasts, and macrophages had strong correlations with all six hub genes and the risk score (**Figure 6A**). Similarly, the proportions of macrophages and fibroblasts predicted by mMCP-counter showed strong correlations with the expression of each hub gene (**Figure 6B**). Furthermore, the risk score (**Figure 6C**) was strongly and positively correlated with macrophages (cor = 0.80, *P* = 3.84e-06), lymphoids (cor = 0.74, *P* < 1e-200), and fibroblasts (cor = 0.69, *P* = 1.34e-04). The results indicated that the expression of each of the six hub genes was strongly correlated with immune infiltration, and the risk score was closely connected with essential immune cells.

### 3.4 Competitive endogenous RNA network

**Figure 7:**
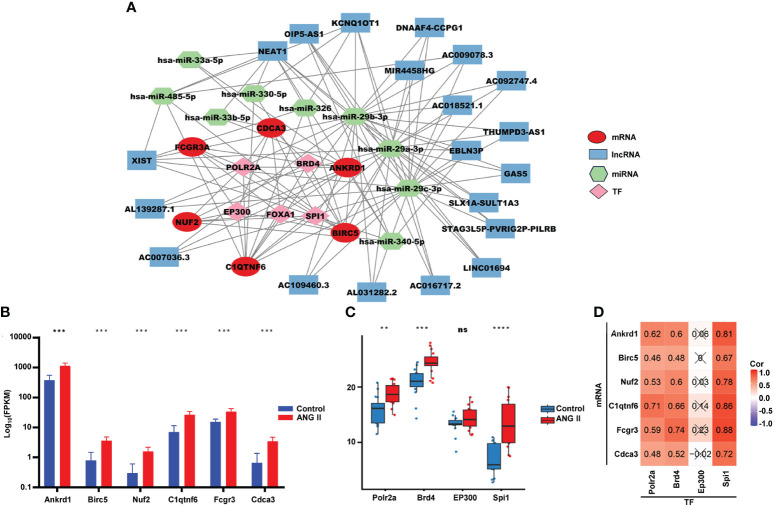
Competitive endogenous RNA network. **(A)** Multifactor-regulated functional network showing the relationships between hub gene mRNA, lncRNA, miRNA, and TFs. **(B)** The expression of six hub genes in RNA-seq. **(C)** The expression of TFs in the control group and the hypertensive-LVH group. **(D)** The correlations between TFs and hub genes. TF, transcription factor. ****P < 0.0001; ***P < 0.001; **P < 0.01; ns, not significantly.

To further investigate the underlying regulatory mechanisms of hub genes, we collected the miRNAs connected to hub genes in ENCORI and the lncRNA correlated with these miRNAs (**Figure 7A**). Five TFs—bromodomain-containing protein 4 (*BRD4*), RNA polymerase II subunit A (*POLR2A*), E1A binding protein p300 (*EP300*), forkhead box A1 (*FOXA1*), and Spi-1 proto-oncogene (*SPI1*)—had regulatory relationships with the hub genes. The expression of hub genes in RNA-seq data is shown in **Figure 7B**, and all six hub genes were found to be upregulated in the hypertensive-LVH group. There were four TFs included in RNA-seq data (**Figure 7C**), and *Polar2a*, *Brd4*, and *Spi1* were overexpressed in the hypertensive-LVH group. Next, the correlations between TFs and hub genes were examined (**Figure 7D**). *Spi1* and *Fcgr3a* had the strongest correlations, each with a correlation coefficient of 0.88. The results showed that the expression of each hub gene was strongly correlated with TFs and indicated the underlying regulatory mechanism.

### 3.5 Validation of the ANG II-infused LVH model

**Figure 8:**
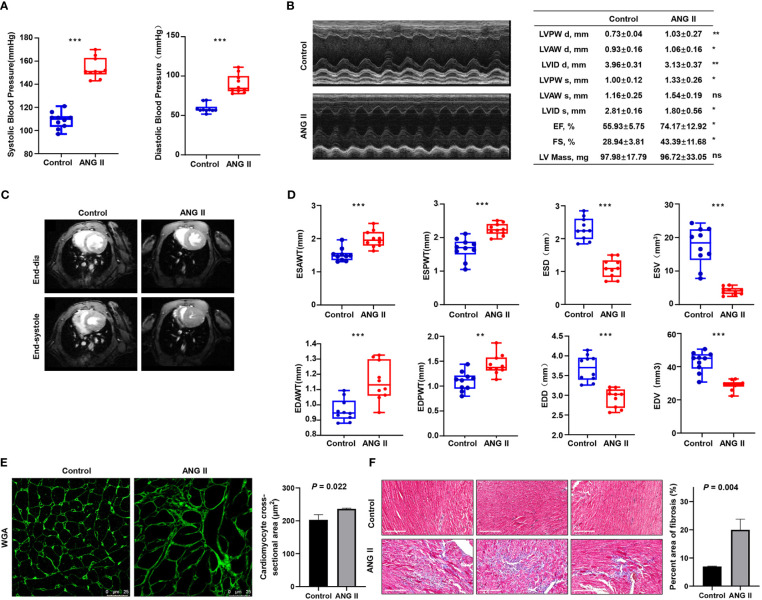
Validation of the ANG II-infused LVH model. **(A)** Blood pressure measured between the control group and ANG II group. **(B)** Representative pictures and results of M-mode echocardiography of the left ventricle in mice with saline infusion or ANG II infusion for 7 days. Data are presented as the mean ± SD for n = 5 mice. **(C)** Representative end‐diastolic and end‐systolic cine MR images of the left ventricle from saline‐treated and ANG II‐treated mice for 7 days. **(D)** Comparison of ESAWT, EDAWT, ESPWT, EDPWT, ESD, EDD, ESV, and EDV between two groups. **(E)** FITC-WGA staining in heart sections of mice with saline or ANG II infusion for 7 days and quantification of the myocyte cross-sectional area (n = 3). **(F)** Masson’s trichrome staining showing collagen deposition in the heart after 7 days of saline or ANG II infusion and quantitative analysis of fibrotic area (Masson trichrome-stained area in light blue normalized to total myocardial area; scale bar = 2 mm). TAC, transverse aortic constriction. ANG II, angiotensin II. LVPW d, left ventricular posterior wall diastolic thickness. LVAW d, left ventricular anterior wall diastolic thickness. LVID d, left ventricular internal diastolic dimension. LVPW s, left ventricular posterior wall systolic thickness. LVAW s, left ventricular anterior wall systolic thickness. LVID s, left ventricular internal systolic dimension. EF, ejection fraction. FS, fractional shortening. LV mass, left ventricular mass. MR, magnetic resonance. ESAWT, end-systolic anterior wall thickness. EDAWT, end diastolic anterior wall thickness. ESPWT, end systolic posterior wall thickness. EDPWT, end diastolic posterior wall thickness. EDD, end-diastolic dimension. ESD, end-systolic dimension. ESV, end-systolic volume. EDV, end-diastole volume. ***P < 0.001; **P < 0.01; *P < 0.05; ns, not significantly.

As ANG II led to the development of hypertension in the LVH mouse model, both systolic and diastolic blood pressure became remarkably elevated in the LVH group compared with the control group (**Figure 8A**). Echocardiography revealed that ANG II hearts had an increased LV wall thickness, diameter, fractional shortening (FS%), and ejection fraction (EF%, **Figure 8B**) compared to the control group, and the LV internal diameter was smaller after Ang II infusion. The CMRI image of the left ventricle in the short axis view demonstrates that the thickness of the left ventricle wall increased significantly in the ANG II treated mice (**Figure 8C**), both in end systole and end diastole. Furthermore, the end-systolic anterior wall thickness (ESAWT), end-diastolic anterior wall thickness (EDAWT), end-systolic posterior wall thickness (ESPWT), and end-diastolic posterior wall thickness (EDPWT) increased in the hypertensive-LVH group, while the end-diastolic dimension (EDD), end-systolic dimension (ESD), end-systolic volume (ESV), and end-diastole volume (EDV) of the left ventricle decreased (**Figure 8D**). To further assess cardiac hypertrophy and function, we used histology with wheat germ agglutinin (WGA) staining to evaluate myocyte size and Masson’s trichrome staining to assess fibrosis condition. Cardiac myocyte size was found to be increased significantly after ANG II infusion in left ventricular tissues (**Figure 8E**). In Masson’ trichrome staining, ANG II infusion significantly increased collagen deposition in left ventricular tissues (**Figure 8F**). Thus, these results suggested that we successfully established a myocardial hypertrophy model in mice.

### 3.6 Validation of immune-related hub genes

Cardiac hypertrophy can be induced by pressure overload and volume overload. Common modeling approaches for pressure overload include transverse aortic constriction (TAC) and ANG II infusion. To validate the expression of immune-related hub genes, GSE186081 was included for external validation of hub gene expression, and the expression data of SHAM-LVH and TAC-LVH groups were used. As shown in **Supplementary Figure 3A**, *Ankrd1*, *Nuf2*, *C1qtnf6*, *Fcgr3*, and *Cdca3* were all upregulated in the TAC-LVH group, and *Birc5* exhibited an increasing trend. In addition, we examined the hub genes in the volume overload model using data from GSE186968 (**Supplementary Figure 3B**). The expression of C1qtnf6 was found to be significantly higher in the volume overload group, while Ankrd1 and Fcgr3 tended to be overexpressed in the hypertrophy group. Inconsistencies found in other genes may be attributed to differences in modeling strategies (27) and the age of selected mice.

**Figure 9:**
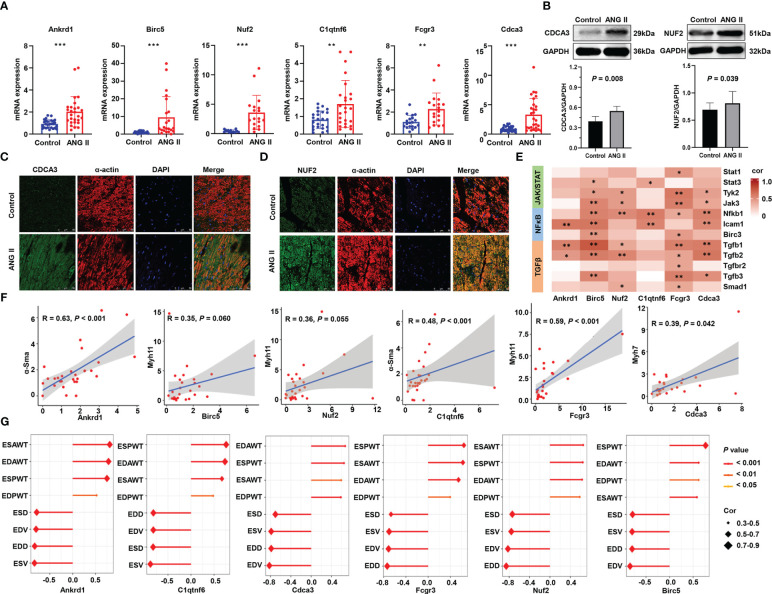
Validation of six immune-related hub gene. **(A)** RNA expression of six hub genes measured by qRT-PCR. **(B)** Western blot analysis of NUF2 and CDCA3 protein levels in hearts and the quantification of protein bands. Data are mean ± SD for n=8 mice. **(C)** Double immunofluorescence analysis of CDCA3 (green) and cardiac myocytes (α-actin, red) in heart samples. **(D)** Double immunofluorescence analysis of NUF2 (green) and cardiac myocytes (α-actin, red) in heart samples. **(E)** Heatmap of the correlation between hub genes and representative pathway markers qualified by q-RT PCR. **(F)** Scatter plots of the correlation of hub genes and myocardial hypertrophy markers. **(G)** Correlation of the structure and function parameters of the left ventricle in mice and the expression of six hub genes. ANG II, angiotensin II. ESAWT, end-systolic anterior wall thickness. EDAWT, end diastolic anterior wall thickness. ESPWT, end systolic posterior wall thickness. EDPWT, end diastolic posterior wall thickness. EDD, end-diastolic dimension. ESD, end-systolic dimension. ESV, end-systolic volume. EDV, end-diastole volume. ***P < 0.001; **P < 0.01; *P < 0.05.

Moreover, RT-qPCR was performed on the left ventricles of ANG-II-induced mice and control mice, with more than 20 samples in each group (**Figure 9A**). All hub genes, namely, *Ankrd1*, *Birc5*, *Nuf2*, *C1qtnf6*, *Fcgr3*, and *Cdca3*, were found to be overexpressed in the hypertensive-LVH group. Considering the existing evidence of *Ankrd1 (* 28, 29), *Birc5* (30, 31), *C1qtnf6 (* 32), and *Fcgr3 (* 33), we particularly focused on the protein expression and the localization of *Nuf2* and *Cdca3*. Western blotting analysis revealed that ANG II treatment resulted in higher level of CDCA3 and NUF2 compared to controls (**Figure 9B**). IF staining showed the co-localization of these two hub proteins with α-actin in ANG II -infused hearts, indicating that cardiac myocytes are the major source of CDCA3 (**Figure 9C**) and NUF2 (**Figure 9D**) in cardiac tissue. In sum, the expression of each hub gene was higher in the LVH group for both the mRNA and protein levels, and CDCA3 and NUF2 were primarily expressed in cardiac myocytes.

To further investigate the roles of immune-related hub genes in indicating dysregulated pathways and myocardial hypertrophy, we performed correlation analysis on the relationship between them. In RNA-seq analysis, the six hub genes had a positive correlation with the signature genes in the JAK/STAT and NFκB pathways and a stronger correlation with genes in the TGF-β pathway (**Supplementary Figure 4**). The results of q-RT PCR verified that *Tgfb1* and *Tgfb2* have a strong correlation with *Ankrd1*, *Birc5*, *Nuf2*, *Fcgr3*, and *Cdca3* (**Figure 9E**), which implies that immune hub genes may promote hypertrophy through the TGF-β pathway. Meanwhile, the hub gene mRNA levels have a strong correlation with myofibroblast differentiation markers (**Figure 9F**). Correlations between hub genes and the structure and function parameters of the left ventricle are displayed in **Figure 9G**. ESAWT, EDAWT, ESPWT, and EDPWT had positive correlations with the expression of each hub gene, whereas EDD, ESD, EDV, and ESV had negative correlations with the expression of each hub gene. These experimental results confirm that immune-related hub genes are strongly related to the hypertrophy pathological process and explain their predictive ability.

### 3.7 Identification of potential small-molecule drugs and molecular docking

**Figure 10:**
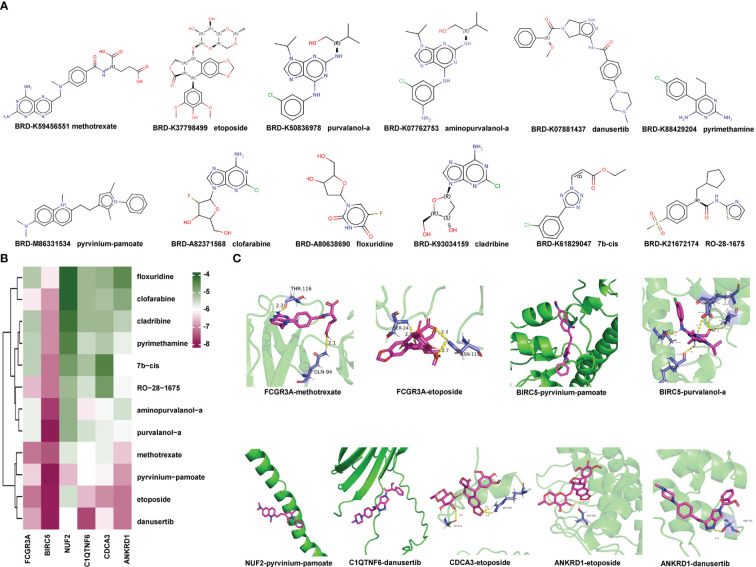
Identification of Potential Small-molecule Drugs and Molecular Docking: **(A)** Structures of the top 12 compounds predicted by CMap website. **(B)** Heatmap displaying the binding energy of small-molecule drug and the hub protein. **(C)** Small-molecule drug docking targets with the lowest binding energy. CMap, Connectivity Map.

To predict small-molecule drugs with the potential to treat LVH, we input the upregulated DEGs into the website CMap. According to the score, 12 small-molecule drugs with the highest negative enrichment scores were identified as potential therapeutic compounds (**Supplementary Table 3**). They were methotrexate, etoposide, pyrvinium-pamoate, pyrimethamine, clofarabine, aminopurvalanol-a, purvalanol-a, floxuridine, cladribine, danusertib, 7b-cis, and RO-28–1675. Their structures were downloaded from CMap and are presented in **Figure 10A**.

Subsequently, we acquired the X-ray diffraction 3D structures of NUF2, BIRC5, and FCGR3A from the RCSB Protein Data Bank (**Supplementary Table 4**). The predicted 3D structures of ANKRD1, CDCA3, and C1QTNF6 were obtained from AlphaFoldDB due to a lack of records in the RCSB Protein Data Bank. The 3D structures of small-molecule drugs were downloaded from PubChem. Then, the software AutoDock Vina was used to calculate the binding affinities between hub proteins and small-molecule drugs. A lower score indicates that lower energy consumption is required for the drug and protein to bind. The predicted binding scores are shown in a heatmap in **Figure 10B**. Protein BIRC5 and compound pyrvinium-pamoate or purvalanol-a had the strongest binding affinities with the lowest absolute AutoDock Vina score of −8.3. Each protein with its lowest binding energy drug is visualized in a 3D structure in **Figure 10C**; **Supplementary Table 5**; the protein is colored green, the small-molecule drug is colored purple, and the yellow dotted lines represent hydrogen bonds. In summary, we identified small-molecule drugs with the potential to treat LVH, and the molecular docking findings indicated that the hub genes may enable clinical drug selection.

## 4 Discussion

LVH involves hypertensive cardiac remodeling and often leads to cardiovascular events such as heart failure and myocardial infarction (34). During this process, cardiomyocytes react to mechanical and neurohormonal signals such as angiotensin II, leading to the activation of specific genes, including c-jun, c-fos, c-myc, atrial natriuretic peptide (ANP), and β-myosin heavy chain (35). Molecular markers such as vascular endothelial growth factor B, growth/differentiation factor 15, and glycoprotein 130 also play crucial roles in myocardial hypertrophy development (36). Despite there being significant progress in the identification of molecular regulators associated with this condition, the intricate nature of hypertrophic remodeling indicates that additional regulatory mechanisms and targets have yet to be discovered. It is important to discover molecular targets in the development of LVH and understand the underlying mechanisms to prevent end-organ damage and adverse outcomes. In this study, we modeled hypertension and LVH through infusing ANG-II *in vivo* and sequenced the left ventricle to identify participating molecules. This paper is the first to combine animal experiments with bioinformatics analysis to identify LVH-related immune genes and comprehensively explore their biological functions.

Pathway enrichment analysis of RNA-seq data revealed that in the hypertensive-LVH group, immune-related processes were upregulated, such as regulation of the inflammatory response and regulation of macrophage migration. This finding is consistent with a previous study showing that immune cell activation is a common feature in hypertrophic responses (26). To further study the key factors of immune system response in LVH, we identified the immune-related hub genes and explored their value in the diagnosis and treatment of LVH.

Machine learning algorithms have greatly facilitated the discovery of biomarkers and new therapeutic targets. In this study, we used WGCNA to identify the immune-related module and employed random forest, SVM-RFE, and LASSO algorithms to screen the immune-related hub genes, which were *Ankrd1*, *Birc5*, *Nuf2*, *C1qtnf6*, *Fcgr3*, and *Cdca3*. Moreover, the expressions of hub genes were validated by both RT-qPCR and an external dataset. In GSE186081, the expressions of *Ankrd1*, *Nuf2*, *C1qtnf6*, *Fcgr3*, and *Cdca3* were upregulated in the left ventricles of the TAC-LVH group in comparison with the SHAM-LVH group. The significantly upregulated relative expression levels of all hub genes from RT-qPCR were consistent with the results from RNA-seq. We also observed a positive correlation between the expression of each hub gene and the LV wall thickness. All these results proved that immune-related hub genes were upregulated and correlated with the progression of LVH.

Ankyrin repeat domain 1 (*ANKRD1*), encoding for the cardiac ankyrin repeat protein (CARP), has been found to be upregulated in hypertrophic stimuli, dilated cardiomyopathy, hypertrophic cardiomyopathy, and heart failure. The roles of *ANKRD1* are complicated, including cardiogenesis, regulation of gene expression, and intracellular signaling (37). Some studies have reported that *ANKRD1* modulates inflammatory responses through NF-κB signaling (38, 39). Research on cell division cycle-associated protein 3 (*CDCA3*), a trigger of mitosis entry 1, mainly focuses on carcinogenesis. Expression of *CDCA3* is a prognostic factor and potential novel therapeutic target in non–small cell lung cancer (40). Although it is upregulated in heart failure, the role of *CDCA3* in cardiovascular disease remains unknown (41). *BIRC5*/survivin, an inhibitor of apoptosis protein family, has been well explored in oncology (42, 43). The upregulation of survivin in myocardial infarction (30) and heart failure (31) has been reported. Moreover, survivin gene therapy attenuates left ventricular systolic dysfunction (44). *Fcgr3* is a member of immunoglobulin Fc receptors. It has been proved that mouse FccRIII is highly expressed on monocytes/macrophages, mast cells, and many other immune cells (45). *Nuf2* has been reported as a prognostic marker and therapeutic target in several types of cancer (46–48) since it becomes elevated following the onset of cancer and promotes tumorigenesis. However, what effect *Nuf2* has on cardiovascular disease is still unclear. *C1qtnf6*/*Ctrp6*, a member of C1q/TNF-related protein (CTRP) family, was demonstrated to increase the synthesis of IL-10 macrophages and promote tumor neovascularization (49). In hypertension, the activation of the CTRP6/ERK/PPARγ axis can alleviate ANG II -induced endothelial dysfunction. Hub genes have exhibited relevance to immunization in previous studies, which verifies the immune-related identification in our research. Although the roles and expression levels of the hub genes in cardiac dysfunction have been reported, this study systematically focused on their immune roles in hypertension-induced LVH and may provide a theoretical basis for the treatment of LVH.

It is noteworthy that macrophages mediate the development of hypertensive LVH. In this study, we found that the proportion of macrophages elevated in the hypertensive-LVH group (as determined by multiple deconvolution algorithms) and the hub genes had strong correlations with the infiltration score of macrophages. The inflammatory role of macrophages in LVH has been reported in many studies. In a TAC mouse model, macrophage depletion for 3 weeks alleviated LVH in both diastolic and systolic wall thickness and LV mass. One study reported that macrophages were recruited into the injured heart by CXC chemokine and subsequently induced cardiac remodeling (50). Since the importance of macrophages in the pathophysiology of hypertension and LVH has been validated, it is meaningful to discover the functions of hub genes in the activation and infiltration of macrophages.

To further explore the underlying mechanisms of hub genes, we utilized ENCORI and hTFtarget to screen the connected miRNA and TFs. In the most strongly connected miRNAs, *miR-133a/b* contributed to muscle or myocardial function. In post-acute myocardial infarction patients, *miR-29b* levels were associated with changes of left ventricular end-diastolic volume (LVEDV) (51, 52). Moreover, we identified five TFs connected to all hub genes, which may have important roles in immune reaction; they are *POLR2A*, *BRD4*, *EP300*, *SPI1*, and *FOXA1*. It has been established that *BRD4* is a central regulator of the pro-fibrotic cardiac fibroblast phenotype, and its inhibitors are promising for therapies in the heart (53). In human arrhythmogenic cardiomyopathy, the activation of the *EP300*-*TP53* pathway is related to altered apical junction structures (54). However, TF *POLR2A* is considered as the overall most stable reference gene across different heart cavities (55). There is little evidence about what roles *SPI1* and *FOXA1* play in cardiac disease. The ceRNA network combining TFs established in this study helped elucidate the potential regulatory mechanisms of hub genes.

Although the development of drugs for LVH treatment has languished, the emergence of novel computational methods may accelerate the discovery of potential drugs and simulation of interactions between proteins and drugs. CMap is a platform to discover drugs with the potential to reverse expression features of diseases. We uploaded the upregulated DEGs in the hypertensive-LVH group and received 12 candidate compounds, which were methotrexate, etoposide, pyrvinium-pamoate, pyrimethamine, clofarabine, aminopurvalanol-a, purvalanol-a, floxuridine, cladribine, danusertib, 7b-cis, and RO-28–1675. Next, we utilized AutoDock Vina to simulate the molecular docking of candidate drugs and hub immune-related proteins and visualize the resulting 3D structure. The common value for selecting potential candidates in drug design is a binding free energy below −6.0 kcal/mol. In our analysis, BIRC5 and compound pyrvinium-pamoate or purvalanol-a had the strongest binding affinities, with the lowest absolute AutoDock Vina score of −8.3. Interestingly, pyrvinium-pamoate, which was previously used as an anthelmintic drug, has been reported to ameliorate myocardial contractile dysfunction in myocardial infarction (56). In cancer, purvalanol-a has been shown to eliminate the activity of survivin, which is coded by *Birc5* (57, 58). Furthermore, methotrexate is used to treat some diseases by reducing the activity of the immune system. Therefore, proteins encoded by hub immune-related genes are potential drug binding sites, and these candidate compounds show promise in the treatment of LVH.

Although our study combined animal model experimentation and bioinformatics analysis, it also had some limitations and shortcomings. First, since heart samples from patients were almost impossible to attain, this study lacked validation in the human left ventricle. Second, the sample size of our study was relatively small, leading to the extremely high AUC of a single gene. However, in clinical application, the utilization of a single gene has a high degree of variability, which may cause deviation between this study and real-world outcomes. Subsequent studies with larger sample sizes are essential to provide strong evidence for confirming the diagnostic value of our genes and signature in the future. Third, the mechanisms of immune-related LVH hub genes in the immune landscape and their abilities to be combined with molecular chemicals demand more sophisticated research. Further experiments *in vitro* and *in vivo* should be conducted in the future.

## 5 Conclusion

By using multiple machine learning algorithms, we screened the hub immune-related genes of ANG-II-induced LVH from RNA-seq data, which were *Ankrd1*, *Birc5*, *Nuf2*, *C1qtnf6*, *Fcgr3*, and *Cdca3*. These hub genes were strongly correlated with fibroblasts and macrophages in immune analysis and were found to be positively associated with LVH in the experimental validation. The immune-related hub genes provide new insights into the intricate pathogenesis of hypertrophic remodeling and hold potential for novel treatments of LVH.

## Data availability statement

All data generated or analyzed during this study are included in this published article and the RNA-seq data has been deposited in the GSE261273 and GSE261275.

## Ethics statement

The animal study was approved by Institutional Animal Care and Use Committee of Institute of Materia Medica, Chinese Academy of Medical Sciences, and Peking Union Medical College. The study was conducted in accordance with the local legislation and institutional requirements.

## Author contributions

MZ: Conceptualization, Investigation, Methodology, Writing – original draft. TL: Methodology, Visualization, Writing – original draft. SL: Data curation, Methodology, Writing – original draft. WG: Validation, Writing – original draft. FZ: Methodology, Software, Writing – original draft. YC: Visualization, Writing – original draft. LY: Validation, Writing – original draft. YH: Formal analysis, Methodology, Writing – original draft. ZY: Validation, Writing – original draft. ZZ: Visualization, Writing – original draft. WZ: Visualization, Writing – original draft. MY: Conceptualization, Funding acquisition, Supervision, Writing – review & editing.

## Glossary

**Table:**

LVH	Left Ventricular Hypertrophy
ANG II	angiotensin II
DEG	Differentially Expressed Gene
TAC	Transverse Aortic Constriction
PCA	Principal Component Analysis
KEGG	Kyoto Encyclopedia of Genes and Genomes
GO	Gene Ontology
GSEA	Gene Set Enrichment Analysis
IF	Immunofluorescence
WGCNA	Weighted Gene Co-expression Network Analysis
SVM-RFE	Support Vector Machine Recursive Feature Elimination
LASSO	Least Absolute Shrinkage and Selection Operator
CMRI	Cardiac Magnetic Resonance Imaging
RT-qPCR	Reverse Transcription-quantitative Polymerase Chain Reaction
GEO	Gene Expression Omnibus database
ECM	Extracellular Matrix
JAK−STAT	Janus kinase-signal transducer and activator of transcription
NF-κB	Nuclear factor kappa B
Ankrd1	Ankyrin repeat domain 1
Birc5	Baculoviral IAP repeat containing 5
Nuf2	NDC80 kinetochore complex component
C1qtnf6	C1q and tumor necrosis factor–related protein 6
Fcgr3	Fc receptor, IgG, low-affinity III
Cdca3	Cell division cycle associated 3
ROC	Receiver Operating Characteristic
AUC	Area Under the Curve
FS	Fractional Shortening
EF	Ejection Fraction
WGA	Wheat Germ Agglutinin.
