# Supplementary material for: Identification of immune-related genes and small-molecule drugs in hypertension-induced left ventricular hypertrophy based on machine learning algorithms and molecular docking
Source: Front Immunol. 2024 Jun 27;15:1351945. doi: 10.3389/fimmu.2024.1351945 (PMC11236603; doi:10.3389/fimmu.2024.1351945)
Supplement: Supplementary file 1 [file DataSheet_1.pdf]

SUPPLEMENTARY MATERIALS

Supplementary Table 1: Sequences of primers in RT-qPCR.

Gene name	Sequence
**Gapdh**	TGGCATTGTGGAAGGGCTCAT<br>CAGCTTTCCAGAGGGGCCAT
**Ankrd1**	GCTGGTAACAGGCAAAAAGAAC<br>CCTCTCGCAGTTTCTCGCT
**Birc5**	GAGGCTGGCTTCATCCACTG<br>CTTTTTGCTTGTTGTTGGTCTCC
**C1qtnf6**	CATCATGGGGATAGCCAGCC<br>GGAGGCCACAGATTCTCCA
**Fcgr3**	CAGAATGCACACTCTGGAAGC<br>GGGTCCCTTCGCACATCAG
**Cdca3**	GAGTAGCAGACCCTCGTTCAC<br>TCTCTACCTGAATAGGAGTGCG
**Nuf2**	TCCCCAGATACAATGTAGCTGA<br>CCGGACTCCATACACTAACTGT
**Stat1**	TCACAGTGGTTCGAGCTTCAG<br>GCAAACGAGACATCATAGGCA
**Stat3**	CAATACCATTGACCTGCCGAT<br>GAGCGACTCAAACTGCCCT
**Tyk2**	AGCCATCTTGGAAGACAGCAA<br>GACTTTGTGTGCGATGTGGAT
**Jak3**	CCATCACGTTAGACTTTGCCA<br>GGCGGAGAATATAGGTGCCTG
**Nfkb1**	GGAGGCATGTTCGGTAGTGG<br>CCCTGCGTTGGATTTCGTG
**Icam1**	GTGATGCTCAGGTATCCATCCA<br>CACAGTTCTCAAAGCACAGCG
**Birc3**	ACGCAGCAATCGTGCATTTTG<br>CCTATAACGAGGTCACTGACGG
**Tgfb1**	CTCCCGTGGCTTCTAGTGC<br>GCCTTAGTTTGGACAGGATCTG

**Tgfb2**	CTTCGACGTGACAGACGCT<br>GCAGGGGCAGTGTAAACTTATT
**Tgfb3**	CCTGGCCCTGCTGAACTTG<br>TTGATGTGGCCGAAGTCCAAC
**Tgfbr2**	CCGCTGCATATCGTCCTGTG<br>AGTGGATGGATGGTCCTATTACA
**Smad1**	GCTTCGTGAAGGGTTGGGG<br>CGGATGAAATAGGATTGTGGGG
**Anp**	GGAGGAGAAGATGCCGGTAGA<br>GCTTCCTCAGTCTGCTCACTCA
**Bnp**	AGCTGCTGGAGCTGATAAGAGAA<br>GTGAGGCCTTGGTCCTTCAA
**α-Sma**	GTCCCAGACATCAGGGAGTAA<br>TCGGATACTTCAGCGTCAGGA
**Myh7**	ACTGTCAACACTAAGAGGGTCA<br>TTGGATGATTTGATCTTCCAGGG
**Myh11**	GCTCGGGACTCAGACTTCAAT<br>GCTGTGGTTGACTCCTGGTG

Supplementary Table 2: Genes included in the weighted gene co-expression network analysis.

Module	Number of genes
Blue	1404
Brown	850
Green	292
Grey	6885
Red	287
Turquoise	4898
Yellow	297
Total	14913

Supplementary Table 3: Information of potential small molecular drugs.

Score	ID	Name	Description	Target
-99.93	BRD-K59456551	methotrexate	Dihydrofolate reductase inhibitor	DHFR, AOX1, FOLR1, TYMS
-99.93	BRD-K37798499	etoposide	Topoisomerase inhibitor	TOP2A, CYP2E1, CYP3A5, TOP2B
-99.93	BRD-M86331534	pyrvinium-pamoate	AKT inhibitor	AR
-99.93	BRD-K88429204	pyrimethamine	Dihydrofolate reductase inhibitor	DHFR, DHFRP1, HEXA, SLC47A1, STAT3
-99.93	BRD-A82371568	clofarabine	Ribonucleoside reductase inhibitor	RRM1, POLA1, RRM2, SLC22A8
-99.93	BRD-K07762753	aminopurvalanol-a	Tyrosine kinase inhibitor	CDK1, CDK2, CDK5, CDK6
-99.93	BRD-K50836978	purvalanol-a	CDK inhibitor	CDK1, CDK2, CDK4, CDK5, CCND1, CCNE1, CSNK1G3, RPS6KA1, SRC
-99.93	BRD-A80638690	floxuridine	DNA synthesis inhibitor	TYMS
-99.93	BRD-K93034159	cladribine	Adenosine deaminase inhibitor	ADA, PNP, POLA1, POLE, POLE2, POLE3, POLE4, RRM1, RRM2, RRM2B
-99.93	BRD-K07881437	danusertib	Aurora kinase inhibitor	AURKA, AURKB, AURKC, FGFR1, NTRK1, RET, BCR, SLK
-99.89	BRD-K61829047	7b-cis	Exportin antagonist	XPO1
-99.89	BRD-K21672174	RO-28-1675	Glucokinase activator	GCK

Supplementary Table 4: Details of the 3D structures of hub proteins.

Hub gene	Protein	Method	Identifier
Ankrd1	Ankyrin repeat domain-containing protein 1	AlphaFoldDB	AF-Q15327-F1
Cdca3	Cell division cycle-associated protein 3	AlphaFoldDB	AF-Q99618-F1
C1qtnf6	Complement C1q tumor necrosis factor-related protein 6	AlphaFoldDB	AF-Q9BXI9-F1
Nuf2	Kinetochore protein Nuf2	X-RAY DIFFRACTION	8G0P
Birc5	Baculoviral IAP repeat-containing protein 5	X-RAY DIFFRACTION	6SHO
FCGR3A	Low affinity immunoglobulin gamma Fc region receptor III-A	X-RAY DIFFRACTION	5YC5

Supplementary Table 5: Binding scores of small molecular drugs and proteins.

	FCGR3A	BIRC5	NUF2	C1QTNF6	CDCA3	ANKRD1
**methotrexate**	**-7.2**	-7.5	-5.7	-6.0	-5.8	-6.5
**etoposide**	**-7.2**	-7.9	-5.6	-6.6	**-7.0**	**-7.2**
**pyrvinium-pamoate**	-6.4	**-8.3**	**-6.6**	-6.0	-6.1	-6.9
**pyrimethamine**	-5.4	-7.0	-4.4	-5.5	-5.3	-5.8
**clofarabine**	-6.2	-6.9	-4.0	-5.1	-5.2	-4.9
**aminopurvalanol-a**	-5.8	-7.7	-5.0	-6.2	-5.9	-5.6
**purvalanol-a**	-5.8	**-8.3**	-5.0	-5.6	-5.8	-5.9
**floxuridine**	-5.3	-6.2	-3.6	-5.1	-5.0	-4.5
**cladribine**	-5.3	-7.0	-4.2	-5.1	-5.1	-5.4
**danusertib**	-6.8	-7.9	-6.0	**-7.6**	-6.2	**-7.2**
**7b-cis**	-5.4	-7.2	-4.7	-5.2	-4.4	-6.0
**RO-28-1675**	-6.6	-7.2	-5.1	-5.6	-4.7	-5.9

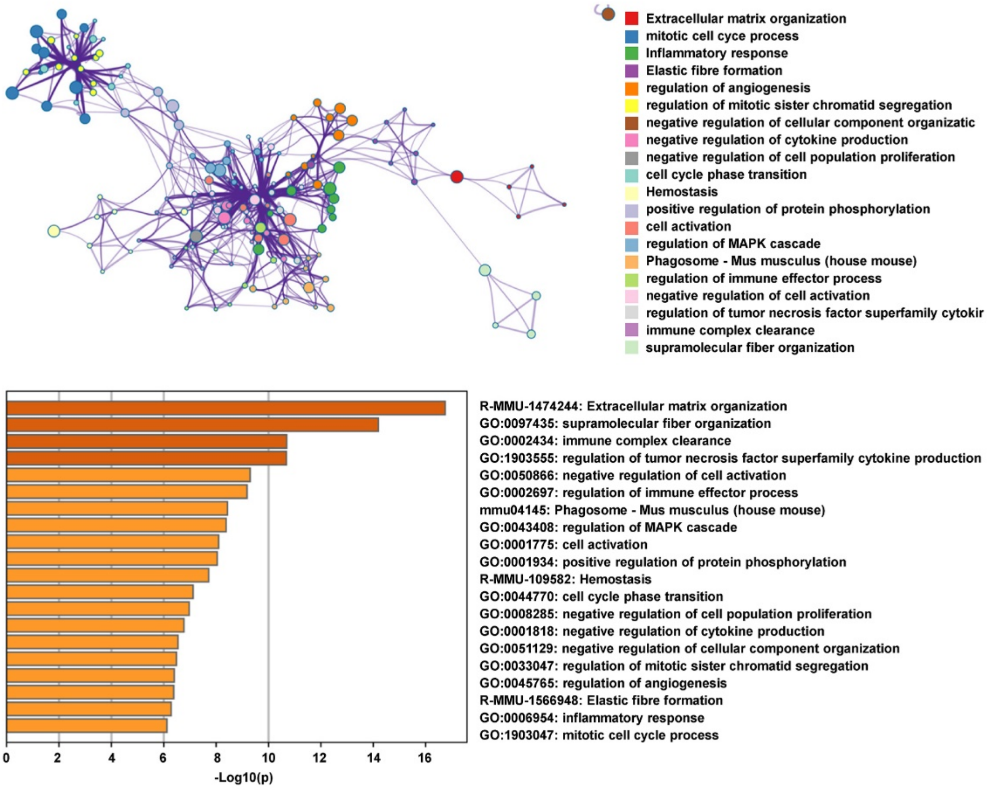

Supplementary Figure 1: Pathway enrichment result in Metascape.

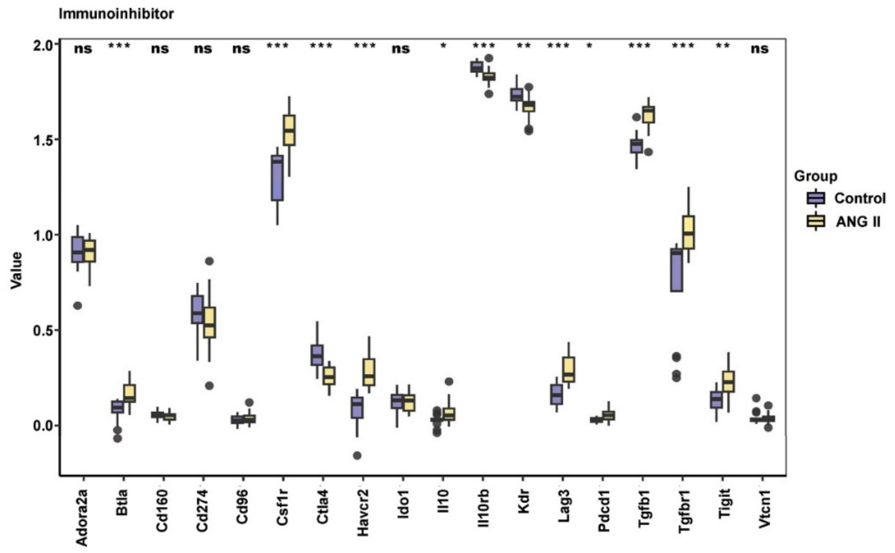

Supplementary Figure 2: Expression of immunoinhibition between the hypertensive-LVH group and the control group. ANG II, angiotensin II. ***$P < 0.001$; **$P < 0.01$; *$P < 0.05$.

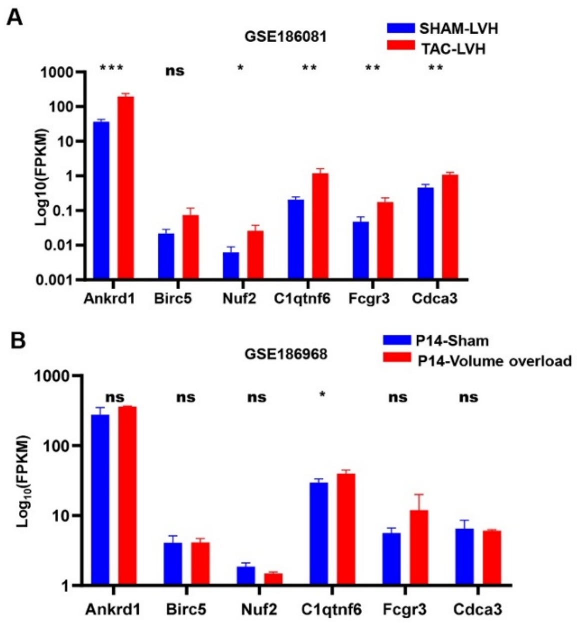

Supplementary Figure 3: Validation of hub immune-related genes in GEO datasets. **(A)** GSE186081 showing the expression of six hub mRNA in cardiac tissues between TAC group and sham group. **(B)** GSE186968 showing the expression of 6 hub mRNA in cardiac tissues between volume overload group and sham group. P14, postnatal day. ****P* < 0.001; ***P* < 0.01; **P* < 0.05; ns, not significantly.

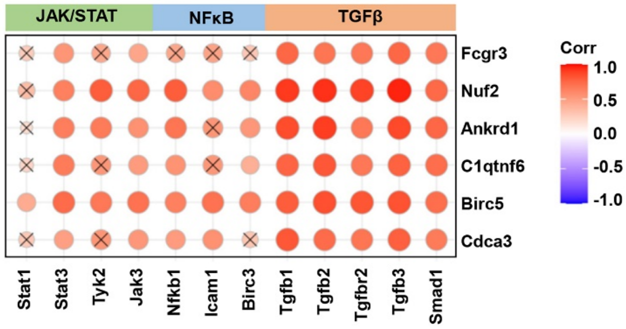

Supplementary Figure 4: The correlation of immune hub genes and representative pathway markers in RNA-seq.
